# Long-Term Sequelae of Severe Acute Kidney Injury in the Critically Ill Patient without Comorbidity: A Retrospective Cohort Study

**DOI:** 10.1371/journal.pone.0121482

**Published:** 2015-03-23

**Authors:** Gijs Fortrie, Susanne Stads, Albert-Jan H. Aarnoudse, Robert Zietse, Michiel G. Betjes

**Affiliations:** 1 Department of Internal Medicine, Division of Nephrology, Erasmus Medical Center, Rotterdam, The Netherlands; 2 Department of Intensive Care, Erasmus Medical Center, Rotterdam, The Netherlands; Bambino Gesù Children's Hospital, ITALY

## Abstract

**Background and Objectives:**

Acute kidney injury (AKI) necessitating renal replacement therapy (RRT) is associated with high mortality and increased risk for end stage renal disease. However, it is unknown if this applies to patients with a preliminary unremarkable medical history. The purpose of this study was to describe overall and renal survival in critically ill patients with AKI necessitating RRT stratified by the presence of comorbidity.

**Design, Setting, Participants, and Measurements:**

A retrospective cohort study was performed, between 1994 and 2010, including all adult critically ill patients with AKI necessitating RRT, stratified by the presence of comorbidity. Logistic regression, survival curve and cox proportional hazards analyses were used to evaluate overall and renal survival. Standardized mortality rate (SMR) analysis was performed to compare long-term survival to the predicted survival in the Dutch population.

**Results:**

Of the 1067 patients included only 96(9.0%) had no comorbidity. Hospital mortality was 56.6% versus 43.8% in patients with and without comorbidity, respectively. In those who survived hospitalization 10-year survival was 45.0% and 86.0%, respectively. Adjusted for age, sex and year of treatment, absence of comorbidity was not associated with hospital mortality (OR=0.74, 95%-CI=0.47-1.15), while absence of comorbidity was associated with better long-term survival (adjusted HR=0.28, 95%-CI = 0.14-0.58). Compared to the Dutch population, patients without comorbidity had a similar mortality risk (SMR=1.6, 95%-CI=0.7-3.2), while this was increased in patients with comorbidity (SMR=4.8, 95%-CI=4.1-5.5). Regarding chronic dialysis dependency, 10-year renal survival rates were 76.0% and 92.9% in patients with and without comorbidity, respectively. Absence of comorbidity was associated with better renal survival (adjusted HR=0.24, 95%-CI=0.07-0.76).

**Conclusions:**

While hospital mortality remains excessively high, the absence of comorbidity in critically ill patients with RRT-requiring AKI is associated with a relative good long-term prognosis in those who survive hospitalization.

## Introduction

Despite improvement in medical care, acute kidney injury (AKI) is a major complication in critically ill patients. Over the past decades a steady increase in incidence of AKI has been reported, while mortality continues to be excessively high [1–5]. The increased incidence of AKI is likely caused by increasing age, a greater burden of comorbidity, pre-existing chronic kidney disease (CKD), the greater use of nephrotoxic drugs and iodine-containing contrast for radiological imaging. Today, AKI occurs in approximately 10–67% of those admitted to the intensive care unit (ICU) [6–13] and in 3–8% renal replacement therapy (RRT) is necessary [6,12]. AKI requiring RRT is associated with a hospital mortality rate about 50–60% and survivors have a substantial risk for end-stage renal disease (ESRD) [3,14–18]. However, an average ICU population is characterized by the heavy burden of comorbidities, which may substantially influence both mortality and renal recovery or even the development of AKI itself [19–21]. In recent literature, the impact of comorbid conditions on renal recovery after AKI and the long-term sequelae is a major topic of discussion [20,22–25]. Given the complex interplay between AKI and comorbidity, it is difficult to determine the true impact of AKI on ESRD and mortality, especially in the long-term. Therefore, overestimation of the risk for these outcomes may occur in subpopulations with lower levels of comorbidity. In particular, in those who are not burdened by any comorbidity. This study describes overall and renal survival in a group of critically ill patients with AKI necessitating RRT stratified by the presence of comorbid conditions.

## Materials and Methods

### Study design and population

A retrospective cohort study was performed including data obtained from patients admitted to a large tertiary care center (Erasmus Medical Center, Rotterdam, The Netherlands). All critically ill patients ≥ 18 years treated with continuous renal replacement therapy (CRRT) between January 1994 and April 2010 were evaluated. Patients with RRT or kidney transplant prior to hospital admission were excluded from analysis. Furthermore, patients in the study population were categorized by the presence of comorbidity in two groups, patients with (comorbid+) and patients without comorbidity (comorbid-). When a patient experienced multiple hospital admissions requiring RRT, only the first hospital admission was used for further analysis. The modalities used for CRRT were continuous arteriovenous haemodialysis (CAVHD) or continuous venovenous haemofiltration (CVVH). Initially, CAVHD was the standard modality for CRRT, which was later gradually replaced by CVVH. CRRT was prescribed by the attending nephrologist and delivered by the haemodialysis nursing team. Intermittent haemodialysis was not performed because most patients in the ICU ward were haemodynamically unstable and the ICU lacks facilities to perform intermittent haemodialysis. The study was approved by the medical ethical review board of the Erasmus Medical Center, which waived the requirement for informed consent, because of its retrospective design.

### Data collection

Data were collected using the hospital electronic patient records (EPR). Detailed clinical and demographic data were collected for patients without comorbidity including primary cause of AKI, type of ICU admission, primary indication for ICU admission, CRRT modality, non-renal SOFA score and number of ICU admission days. Furthermore, at hospital admission, at start of CRRT and at hospital discharge serum creatinine values were collected. Baseline renal function was not known in the majority of patients, as they were not under medical care prior to hospital admission. Given the uncertain relation between serum creatinine concentrations and renal function at hospital admission and start of CRRT we only calculated the estimated glomerular filtration rate (eGFR) at hospital discharge. To determine whether a patient reached ESRD requiring RRT after hospital discharge we used data from the RENINE Foundation. This foundation manages a Dutch national database containing all patients treated with RRT for at least 3 months and therefore considered chronically dependent on RRT.

### Definitions

The patient records were used to identify whether patients were known with malignancy, solid organ transplantation, intravenous drug abuse and pre-existing chronic diseases such as CKD, hypertension, diabetes mellitus, liver failure, cardiovascular diseases, autoimmune diseases, chronic obstructive pulmonary disease (COPD), connective tissue diseases and chronic infectious diseases like HIV and hepatitis. Patients without one of these conditions were categorized in the comorbid- group, while patients with one or more of these conditions were categorized in the comorbid+ group. The primary cause of AKI was categorized as: sepsis, ischemia, drug-associated and other. Sepsis was defined in accordance to the Surviving Sepsis Campaign International Guidelines [26]. Ischemia was defined as AKI due to hypotension and pre-renal kidney failure. All patients suffering from AKI due to drugs, contrast and other substances that are nephrotoxic were categorized in the drug-associated group. Patients that experienced an episode of AKI due to any other cause then aforementioned, consisting rhabdomyolysis and glomerulonephritis were categorized in the “other” group. Indications for ICU admittance were categorized as: sepsis, postoperative, traumatic injury, intoxication and other. A postoperative ICU indication was defined as the need for ICU admission for treatment and monitoring due to perioperative haemodynamic instability. Reasons for surgery included acute pancreatitis, stomach and bowel perforations, an intra abdominal abscess and a total hip prosthesis. For estimation of the GFR we used the modified diet in renal diseases (MDRD) formula adjust for age and sex [27].

### Study outcomes

Primary study outcomes were overall and renal survival stratified by the presence of comorbidity. Overall survival was divided into hospital mortality and survival after hospital discharge. Renal survival was defined as the time until the need for chronic RRT. Long-term overall and renal survival rates were presented at 1, 5 and 10 years after discharge. In addition, long-term overall survival was compared to the predicted survival in the Dutch population. Secondary, patients without comorbidity were evaluated for independent predictors associated with hospital mortality. Due to the low number of events in patients without comorbidity predictors for overall and renal survival were not evaluated.

### Statistical analysis

Continuous parameters were expressed as median and interquartile range. Categorical parameters were expressed as number and percentage. Logistic regression analysis adjusted for age, sex and year of treatment was performed to compare hospital mortality in patients with and without comorbidity. In patients without comorbidity, logistic regression analysis was performed to determine independent predictors for hospital mortality. Parameters with a p-value ≤ 0.1 reported by univariable analysis were considered eligible for multivariable analysis. Irrespective of p-value the variables age, sex and year of treatment were included in multivariable analysis. Overall and renal survival after hospital discharge stratified by presence of comorbidity was evaluated by Kaplan-Meier analysis. Log-rank test was used to analyze crude differences pooled over strata and cox proportional hazards analysis was used to adjust for age, sex and year of treatment. The standardized mortality ratio (SMR) was calculated by comparing mortality after hospital discharge with the expected mortality in the general Dutch population. The SMR is the ratio of observed to expected number of deaths. The expected number of deaths is calculated by multiplying the total number of years lived by patients in the study population for each calendar period in each age and sex category by the age and sex specific mortality rates of the Dutch population for each calendar period. A two-tailed p-value ≤ 0.05 was considered significant. Analyses were performed using statistical software SPSS, version 20.0 for Mac (SPSS Inc., an IBM company, Chicago, IL, USA) and GraphPad Prism version 5.0a for Mac (GraphPad Software, La Jolla, CA, USA)

## Results

### Study population

**Fig 1:**
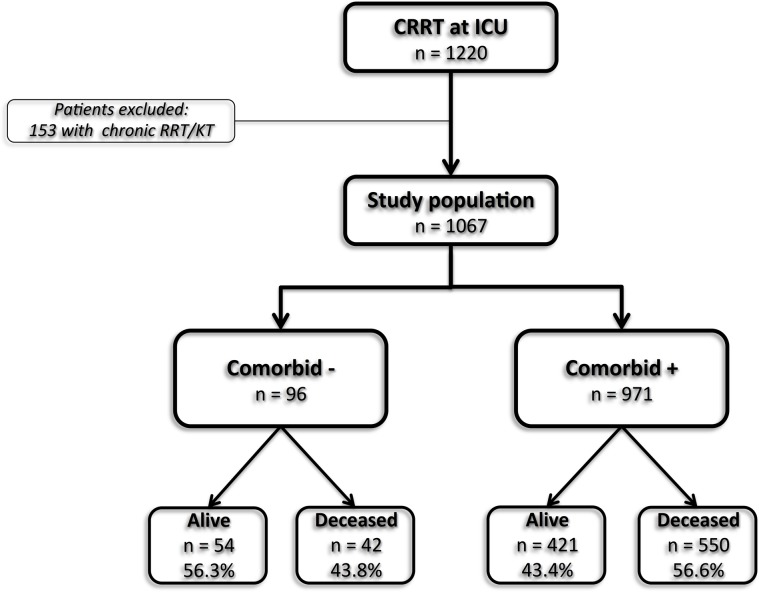
Flowchart of inclusion and hospital mortality stratified by presence of comorbidity. (C)RRT: (continuous) renal replacement therapy, ICU: intensive care unit, KT: kidney transplant.

A total of 1220 patients treated with CRRT during ICU admission were evaluated during the study period. After exclusion of 153 patients on chronic RRT or with a kidney transplant the study population included 1067 patients of which 96 (9.0%) had no comorbidity (Fig. 1). Clinical characteristics of the patients without comorbidity are presented in Table 1. The median age was 45 years and 55.2% of the patients were of male gender. The most common cause of AKI was sepsis (58.3%) followed by ischemia (25.0%). In the largest group of patients sepsis (37.5%) was also the most frequently indication for ICU admission followed by trauma (31.3%). CAVHD or CVVH were used in 39 (40.6%) and 57 (59.4%) of the patients as modality for CRRT. The median non-renal SOFA score at ICU admission was 10 points and only available in 50 patients. The median serum creatine at hospital admission and start of CRRT were 177 and 427 μmol/L, respectively, and the median length of ICU stay was 21 days.

**Table 1:** Clinical and demographical characteristics of 96 patients without comorbidity treated with CRRT in the ICU.

Characteristic		
Age in years (interquartile range)		45 (35–60)
Male sex (%)		53 (55.2)
Cause of AKI (%)		
	Sepsis	56 (58.3)
	Ischemia	24 (25.0)
	Drug-associated	9 (9.4)
	Other	7 (7.3)
Surgical admission (%)		64 (66.7)
Indication for ICU admission (%)		
	Sepsis	36 (37.5)
	Post-operative	12 (12.5)
	Intoxication	10 (10.4)
	Trauma	30 (31.3)
	Other	8 (8.3)
CRRT modality (%)		
	CAVHD	39 (40.6)
	CVVH	57 (59.4)
Non-renal SOFA score (interquartile range)*		10 (8–13)
Serum creatinine in μmol/L (interquartile range)		
	Hospital admission	177 (94–350)
	Start CRRT	427 (298–569)
Days of ICU stay (interquartile range)		21 (11–38)

Categorical variables are expressed as number and percentage; continuous variables are expressed as median and interquartile range. AKI: acute kidney injury; CAVHD: continuous arteriovenous haemodialysis; CRRT: continuous renal replacement therapy; CVVH: continuous venovenous hemofiltration; ICU: intensive care unit; SOFA: sequential organ failure assessment

* Score available in 50 cases

### Hospital mortality

In the group without comorbidity 42 (43.8%) patients deceased during hospitalization compared to 550 (56.6%) of those with comorbidity, respectively (P = 0.02). Adjusted for age, sex and year of treatment, patients without comorbidity had a similar hospital mortality risk (Odds ratio [OR] = 0.74, 95% confidence interval [CI] = 0.47–1.15). In subgroup analysis on patients without comorbidity, univariable analysis identified several clinical variables associated with hospital mortality presented in Table 2. Only serum creatinine at start of CRRT (OR = 0.96, 95%-CI = 0.93–0.99) and length of ICU stay (OR = 0.96, 95%-CI = 0.94–0.99) remained associated with hospital mortality after multivariable logistic regression analysis.

**Table 2:** Univariable and multivariable analysis of characteristics associated with hospital mortality in patients without comorbidity.

		Univariable Analysis		Multivariable Analysis	
		OR (95%-CI)	P-value	OR (95%-CI)	P-value
Age in years		1.02 (0.99–1.05)	0.13	1.03 (1.00–1.07)	0.08
Male sex		1.37 (0.60–3.09)	0.45	2.46 (0.67–9.02)	0.18
Surgical admission		1.00 (0.43–2.35)	1.00	-	-
Indication for ICU admission					
	Sepsis	1		1	
	Post-operative	0.50 (0.13–1.96)	0.32	1.11 (0.21–5.85)	0.90
	Intoxication	0.43 (0.10–1.93)	0.27	0.49 (0.09–2.73)	0.42
	Trauma	1.14 (0.43–3.02)	0.79	3.17 (0.81–12.37)	0.10
	Other	0.14 (0.02–1.28)	0.08	0.44 (0.04–5.46)	0.52
CVVH as CRRT modality		0.60 (0.26–1.36)	0.22	-	-
Non-renal SOFA score*		1.07 (0.93–1.24)	0.36	-	-
Serum creatinine in μmol/L per 10 points					
	Hospital admission	0.99 (0.97–1.01)	0.40	-	-
	Start CRRT	0.98 (0.96–1.00)	0.04	0.96 (0.93–0.99)	0.01
Days of ICU stay		0.98 (0.96–0.99)	0.01	0.96 (0.94–0.99)	0.003
Year of treatment		0.96 (0.88–1.04)	0.31	0.94 (0.83–1.05)	0.28

CRRT: continuous renal replacement therapy; CVVH: continuous venovenous hemofiltration; ICU: intensive care unit; SOFA: sequential organ failure assessment

*Score available in 50 cases

### Survival after hospital discharge

**Fig 2:**
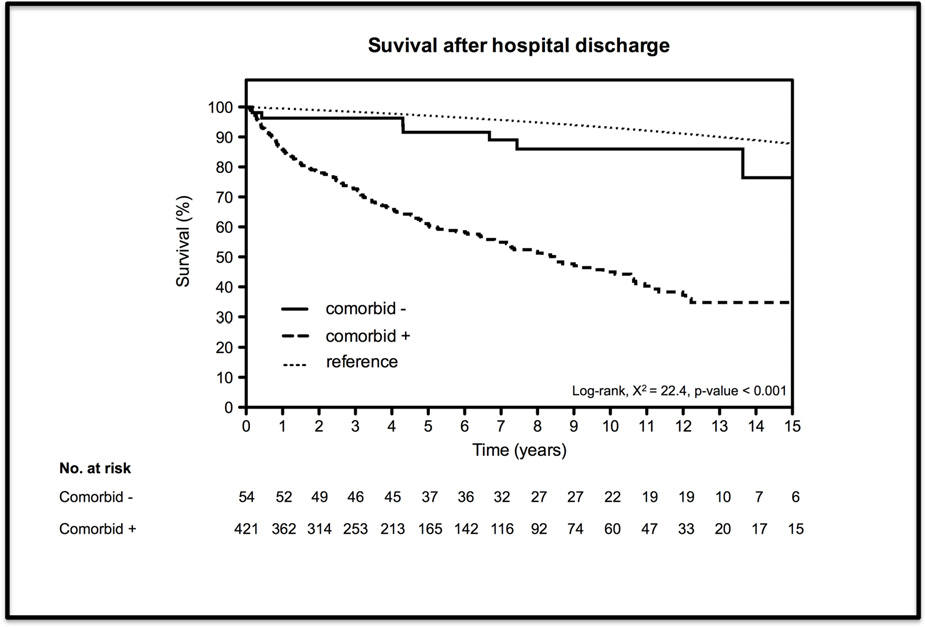
Kaplan-Meier curves for overall survival after hospital discharge stratified by comorbidity. The reference curve represents the predicted survival in the Dutch population matched for age, sex and calendar period to patients without comorbidity.

In total, 475 patients left the hospital alive of which 54 had no comorbidity. Median follow-up time was 4.4 years (2.1–8.0). In general, the percentage of survival at 1, 5 and 10 years in those that survived hospitalization was 87.2%, 64.7% and 50.4%, respectively. Stratified by presence of comorbidity survival rates were 85.7%, 61.1% and 45.0% compared to 96.3%, 91,6% and 86.0% in patients with and without comorbidity, respectively (Table 3). Survival curves are presented in Fig. 2 and log-rank test comparing both groups showed a crude significant difference in survival (p < 0.001). Adjusted for age, sex and year of treatment, patients without comorbidity had a significant better survival rate (Hazard-ratio [HR] = 0.28, 95%-CI = 0.14–0.58). Compared to the predicted survival in the Dutch population patients without comorbidity had a similar mortality risk (SMR = 1.6, 95%-CI = 0.7–3.2), while this risk was significantly increased in patients with comorbidity (SMR = 4.8, 95%-CI = 4.1–5.5) (Table 4). A reference curve, shown in Fig. 2, represents the predicted survival in the Dutch population matched for age, sex and calendar period to patients without comorbidity.

**Table 3:** Overall and renal survival of patients that survived hospital admission.

	Overall survival (%)			Renal survival (%)		
	1 yr	5 yr	10 yr	1 yr	5 yr	10 yr
**Comorbid +**	85.7	61.1	45.0	85.8	82.9	76.0
**Comorbid -**	96.3	91.6	86.0	96.3	96.3	92.9

Data are given as percentage of cumulative overall survival and renal survival at 1, 5 and 10 years stratified by the presence of comorbidity.

**Table 4:** Standardized mortality ratio analysis in patients that survived hospital admission.

	Sex	No. in group	No. of deaths	Person years	SMR (95%-CI)	P-value
**Comorbid +**	Male	286	133	1441.8	4.3 (3.6–5.6)	<0.001
	Female	135	59	691.8	6.5 (4.9–8.4)	<0.001
	**Overall**	**421**	**192**	**2133.6**	**4.8 (4.1–5.5)**	**<0.001**
**Comorbid -**	Male	29	6	255.6	1.7 (0.6–3.6)	0.15
	Female	25	2	206.9	1.5 (0.2–5.2)	0.40
	**Overall**	**54**	**8**	**462.4**	**1.6 (0.7–3.2)**	**0.10**

### Renal function and renal survival after hospital discharge

**Fig 3:**
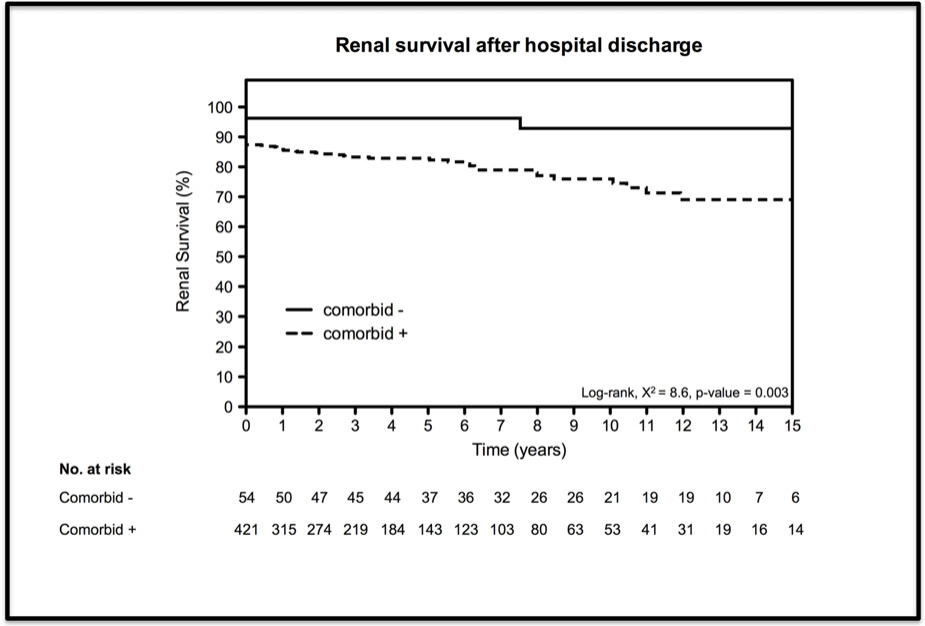
Kaplan-Meier curves for renal survival stratified by comorbidity. Defined as years after discharge until chronic replacement therapy is initiated, censored for death.

At time of hospital discharge 55 (11.6%) of all patients were dialysis dependent of which 53 (12.6%) and 2 (3.7%) with and without comorbidity, respectively (p = 0.07). Renal survival rates at 1, 5 and 10 years were, respectively, 85.8%, 82.9% and 76.0% versus 96.3%, 96.3% and 92.9% in patients with and without comorbidity (Table 3). Renal survival curves stratified by comorbidity are presented in Fig. 3 and log-rank test comparing both groups showed a crude significant difference in renal survival (p = 0.003). Adjusted for age, sex and year of treatment patients without comorbidity had a significant better renal survival rate (HR = 0.24, 95%-CI = 0.07–0.76). At hospital discharge, grouped by eGFR, 28 (51.9%) patients without comorbidity had an eGFR ≥ 90, 9 (16.7%) an eGFR = 60–89, 9 (16.7%) an eGFR = 30–59, 3 (5.6%) an eGFR = 15–29 and 5 (9.3%) patients left the hospital with an eGFR < 15 ml/min/1.73m^2^.

## Discussion

To the best of our knowledge this is the first study to describe overall and renal survival in a group of critically ill patients with AKI necessitating RRT stratified by the presence of comorbid conditions. We demonstrated that patients without comorbidity constitute a minority of the ICU population, as only 9% of all ICU patients treated with RRT were not burdened with relevant pre-existing diseases. Although, hospital mortality in this group was still high, the overall and renal survival after hospital discharge was relatively good with 10-year survival rates of 86% and 93%, respectively. In particular the fact that we could not identify a difference in long-term mortality risk compared to the predicted mortality in the Dutch population is of interest.

### Hospital mortality and associated risk factors

In the overall study population the hospital mortality rate was 55.5%, which is in accordance with the results of previous studies [3]. We demonstrated that patients without comorbidity had a crude decreased risk for mortality, but contrary to what we expected, no significant difference was found after adjusting for age, sex and year of treatment. Given the fact that a trend towards better survival persisted, it is possible that the lack of statistical significance is due to the small population of patients without comorbidity. For instance, a recent study by *Ostermann and Chang* [28] evaluating a large cohort of patients (n = 1847) treated with RRT in the ICU, reported that the presence of one or more comorbidities was associated with increased ICU mortality. In spite of these results the risk for mortality during hospital admission in the critically ill patient without comorbidity remains excessively high. Multivariable analysis in patients without comorbidity revealed that serum creatinine at start of CRRT and length of ICU stay were associated with hospital mortality. Interestingly, a higher serum creatinine at start of RRT was associated with lower mortality. This finding is in accordance with previous studies, which demonstrated that an increase in RIFLE criteria was associated with higher mortality [29,30], while an absolute higher serum creatinine at time of diagnosis of AKI [29] or start of RRT [31,32] was associated with lower mortality. A hypothetical explanation for this observation is that low serum creatinine levels at start of RRT reflect poor clinical condition rather than better renal function as these patients may have had less muscle mass and/or could have been more fluid overloaded.

### Long-term survival after hospitalization

The results on long-term survival presented in the overall study population are in accordance with the results of previous studies [33–38]. In contrast to hospital survival, we demonstrated that there was a great difference in survival after hospitalization in favour of those without comorbidity (adjusted HR = 0.28). Because this is the first study evaluating long-term mortality after AKI stratified by presence or absence of comorbidity it is not possible to directly compare these results to previous studies. Furthermore, studies that reported survival rates after for instance 5 or 10 years are scarce. Two studies that evaluated long-term mortality after AKI requiring RRT reported overall survival rates after 5 years of 15.5 to 35.5%, including those who died during hospitalization. These results are in accordance to the survival rate of 26.5% in the group with comorbidity in our study, including those who died during hospitalization. In patients without comorbidity this was 51.5%. Interestingly, patients without comorbidity had a similar long-term mortality risk as predicted in the Dutch population. However, a trend towards a higher mortality risk was reported, and the lack of statistical significance could be the result small study size, which implies that future studies with a larger sample size are warranted.

### Renal survival after hospitalization

At hospital discharge 11.6% of all patients were dependent on RRT, which is about average compared to results of previous studies that reported a percentage ranging from 0 to 32% [15,16,33,39,40]. Our result demonstrated that in patients without comorbidity only 3,7% patients left the hospital dependent on RRT, which is low compared to most of the aforementioned studies. However, *Schiffl et al*. reported that none of the 425 critically ill patients included in their study reached dialysis dependence at hospital discharge [39]. Interestingly, this is the only study that excluded all patients with a preliminary impaired renal function. These results suggest that in particular an impaired renal function prior AKI is an important risk factor for dialysis dependence thereafter. Furthermore, in a previous study of our research group we found that in the presence of chronic kidney disease no other comorbid condition was significantly associated with the need for RRT at hospital discharge in patients surviving AKI requiring RRT [16]. After hospital discharge only one more patient became chronic dialysis dependent after 7.5 years of follow-up. This 33-year-old male patient was admitted to the ICU after a severe trauma (motor accident) and left the hospital with an eGFR of 28 ml/min/1.73m², which slowly decreased towards ESRD necessitating dialysis. An explanation for the high renal survival rate reported in our study is the low number of patients that left the hospital with an impaired renal function, which is, as reported by *Stads et al*., an important predictor for progression towards ESRD requiring RRT [38].

### Limitations

There are certain limitations to our study that should be taken into consideration before interpretation of the results. First, the single center retrospective design has its inherent drawbacks and does not offer the possibility to establish causality and it is not known if the results can be generalized to other ICU populations. Second, the population of patients without comorbidity was rather small, which results in a lack of statistical power. Third, our study included patients over a period of 16 years and it is likely that patterns of referral to and treatment in the ICU have changed over time. Therefore, the year of therapy was included in all multivariable analyses to adjust for possible confounding. Third, it is possible that patients without comorbidity had chronic renal impairment before hospital admission, which could bias the results of our study. However, even if some patients with unknown chronic renal impairment were included it would strengthen our conclusion, because the long-term prognosis in patients without comorbidity would be even better. Fourth, it was not possible to collect information on progressive loss of renal function besides progression towards ESRD or renal function at time of hospital discharge. Thus, it is possible that besides the low number of patients that progressed towards ESRD there actually was deterioration in renal function. Fifth, besides modality of CRRT, no further detailed information was available including type of dialysis access, type of anticoagulation regime, subsequent complications, etc. However, given these limitations, the results of this study are of interest as the presence or absence of comorbidity seems to have a substantial effect on the prognosis of the critically ill patient and this study offers an interesting perspective on such a complex syndrome as AKI.

## Conclusions

The results of our study are indicative that the absence of comorbidity in critically ill patients with RRT-requiring AKI does not have a major impact on hospital mortality but is associated with a relatively good long-term survival rate and infrequent progression to ESRD. However, given the aforementioned limitations, future prospective studies with a large sample size are warranted before firm conclusions can be drawn.

## References

[1] Susantitaphong P, Cruz DN, Cerda J, Abulfaraj M, Alqahtani F, Koulouridis I, et al World incidence of AKI: a meta-analysis. Clin J Am Soc Nephrol. 2013;8:1482–1493. 10.2215/CJN.00710113 23744003 PMC3805065

[2] Waikar SS, Curhan GC, Wald R, McCarthy EP, Chertow GM. Declining mortality in patients with acute renal failure, 1988 to 2002. J Am Soc Nephrol. 2006;17:1143–1150. 16495376 10.1681/ASN.2005091017

[3] Ympa YP, Sakr Y, Reinhart K, Vincent JL. Has mortality from acute renal failure decreased? A systematic review of the literature. Am J Med. 2005;118:827–832. 16084171 10.1016/j.amjmed.2005.01.069

[4] Xue JL, Daniels F, Star RA, Kimmel PL, Eggers PW, Molitoris BA, et al Incidence and mortality of acute renal failure in Medicare beneficiaries, 1992 to 2001. J Am Soc Nephrol. 2006;17:1135–1142. 16495381 10.1681/ASN.2005060668

[5] Hsu CY, McCulloch CE, Fan D, Ordonez JD, Chertow GM, Go AS. Community-based incidence of acute renal failure. Kidney Int. 2007;72:208–212. 17507907 10.1038/sj.ki.5002297 PMC2673495

[6] Cruz DN, Bolgan I, Perazella MA, Bonello M, de Cal M, Corradi V, et al North East Italian Prospective Hospital Renal Outcome Survey on Acute Kidney Injury (NEiPHROS-AKI): targeting the problem with the RIFLE Criteria. Clin J Am Soc Nephrol. 2007;2:418–425. 17699446 10.2215/CJN.03361006

[7] Thakar CV, Christianson A, Freyberg R, Almenoff P, Render ML. Incidence and outcomes of acute kidney injury in intensive care units: a Veterans Administration study. Crit Care Med. 2009;37:2552–2558. 10.1097/CCM.0b013e3181a5906f 19602973

[8] Joannidis M, Metnitz B, Bauer P, Schusterschitz N, Moreno R, Druml W, et al Acute kidney injury in critically ill patients classified by AKIN versus RIFLE using the SAPS 3 database. Intensive Care Med. 2009;35:1692–1702. 10.1007/s00134-009-1530-4 19547955

[9] Ostermann M, Chang RW. Challenges of defining acute kidney injury. Qjm. 2011;104:237–243. 10.1093/qjmed/hcq185 20934982

[10] Ostermann M, Chang R. Correlation between the AKI classification and outcome. Crit Care. 2008;12:R144 10.1186/cc7123 19019254 PMC2646305

[11] Bagshaw SM, George C, Bellomo R. A comparison of the RIFLE and AKIN criteria for acute kidney injury in critically ill patients. Nephrol Dial Transplant. 2008;23:1569–1574. 10.1093/ndt/gfn009 18281319

[12] Garzotto F, Piccinni P, Cruz D, Gramaticopolo S, Dal Santo M, Aneloni G, et al RIFLE-based data collection/management system applied to a prospective cohort multicenter Italian study on the epidemiology of acute kidney injury in the intensive care unit. Blood Purif. 2011;31:159–171. 10.1159/000322161 21228585

[13] Hoste EA, Clermont G, Kersten A, Venkataraman R, Angus DC, De Bacquer D, et al RIFLE criteria for acute kidney injury are associated with hospital mortality in critically ill patients: a cohort analysis. Crit Care. 2006;10:R73 16696865 10.1186/cc4915 PMC1550961

[14] Uchino S, Kellum JA, Bellomo R, Doig GS, Morimatsu H, Morgera S, et al Acute renal failure in critically ill patients: a multinational, multicenter study. Jama. 2005;294:813–818. 16106006 10.1001/jama.294.7.813

[15] Wald R, Deshpande R, Bell CM, Bargman JM. Survival to discharge among patients treated with continuous renal replacement therapy. Hemodial Int. 2006;10:82–87. 16441832 10.1111/j.1542-4758.2006.01179.x

[16] Fortrie G, Stads S, de Geus HR, Groeneveld AB, Zietse R, Betjes MG. Determinants of renal function at hospital discharge of patients treated with renal replacement therapy in the intensive care unit. J Crit Care. 2013;28:126–132. 10.1016/j.jcrc.2012.10.013 23265287

[17] Coca SG, Yusuf B, Shlipak MG, Garg AX, Parikh CR. Long-term risk of mortality and other adverse outcomes after acute kidney injury: a systematic review and meta-analysis. Am J Kidney Dis. 2009;53:961–973. 10.1053/j.ajkd.2008.11.034 19346042 PMC2726041

[18] Coca SG, Singanamala S, Parikh CR. Chronic kidney disease after acute kidney injury: a systematic review and meta-analysis. Kidney Int. 2012;81:442–448. 10.1038/ki.2011.379 22113526 PMC3788581

[19] Kellum JA, Bellomo R, Ronco C. Kidney attack. Jama. 2012;307:2265–2266. 10.1001/jama.2012.4315 22572776

[20] Chawla LS, Kimmel PL. Acute kidney injury and chronic kidney disease: an integrated clinical syndrome. Kidney Int. 2012;82:516–524. 10.1038/ki.2012.208 22673882

[21] Goldstein SL, Chawla LS. Renal angina. Clin J Am Soc Nephrol. 2010;5:943–949. 10.2215/CJN.07201009 20299370

[22] Rifkin DE, Coca SG, Kalantar-Zadeh K. Does AKI truly lead to CKD? J Am Soc Nephrol. 2012;23:979–984. 10.1681/ASN.2011121185 22460531 PMC3358766

[23] Hsu CY. Yes, AKI truly leads to CKD. J Am Soc Nephrol. 2012;23:967–969. 10.1681/ASN.2012030222 22499588

[24] James MT, Wald R. AKI: not just a short-term problem? Clin J Am Soc Nephrol. 2014;9:435–436. 10.2215/CJN.00500114 24526743 PMC3944751

[25] Cohen SD, Kimmel PL. Long-term sequelae of acute kidney injury in the ICU. Curr Opin Crit Care. 2012;18:623–628. 10.1097/MCC.0b013e328358d3f5 22941209

[26] Dellinger RP, Levy MM, Rhodes A, Annane D, Gerlach H, Opal SM, et al Surviving sepsis campaign: international guidelines for management of severe sepsis and septic shock: 2012. Crit Care Med. 2013;41:580–637. 10.1097/CCM.0b013e31827e83af 23353941

[27] Levey AS, Bosch JP, Lewis JB, Greene T, Rogers N, Roth D. A more accurate method to estimate glomerular filtration rate from serum creatinine: a new prediction equation. Modification of Diet in Renal Disease Study Group. Ann Intern Med. 1999;130:461–470. 10075613 10.7326/0003-4819-130-6-199903160-00002

[28] Ostermann M, Chang RW. Correlation between parameters at initiation of renal replacement therapy and outcome in patients with acute kidney injury. Crit Care. 2009;13:R175 10.1186/cc8154 19889205 PMC2811955

[29] Cruz DN, Bolgan I, Perazella MA, Bonello M, de Cal M, Corradi V, et al North East Italian Prospective Hospital Renal Outcome Survey on Acute Kidney Injury (NEiPHROS-AKI): targeting the problem with the RIFLE Criteria. Clin J Am Soc Nephrol. 2007;2:418–425. 17699446 10.2215/CJN.03361006

[30] Ostermann M, Chang R, Riyadh ICUPUG. Correlation between the AKI classification and outcome. Crit Care. 2008;12:R144 10.1186/cc7123 19019254 PMC2646305

[31] Cerda J, Cerda M, Kilcullen P, Prendergast J. In severe acute kidney injury, a higher serum creatinine is paradoxically associated with better patient survival. Nephrol Dial Transplant. 2007;22:2781–2784. 17597091 10.1093/ndt/gfm395

[32] Aldawood A. Outcome and prognostic factors of critically ill patients with acute renal failure requiring continuous renal replacement therapy. Saudi J Kidney Dis Transpl. 2010;21:1106–1110. 21060181

[33] Bagshaw SM, Laupland KB, Doig CJ, Mortis G, Fick GH, Mucenski M, et al Prognosis for long-term survival and renal recovery in critically ill patients with severe acute renal failure: a population-based study. Crit Care. 2005;9:R700–709. 16280066 10.1186/cc3879 PMC1414056

[34] Carl DE, Grossman C, Behnke M, Sessler CN, Gehr TW. Effect of timing of dialysis on mortality in critically ill, septic patients with acute renal failure. Hemodial Int. 2010;14:11–17. 10.1111/j.1542-4758.2009.00407.x 20377649

[35] Korkeila M, Ruokonen E, Takala J. Costs of care, long-term prognosis and quality of life in patients requiring renal replacement therapy during intensive care. Intensive Care Med. 2000;26:1824–1831. 11271091 10.1007/s001340000726

[36] Morgera S, Kraft AK, Siebert G, Luft FC, Neumayer HH. Long-term outcomes in acute renal failure patients treated with continuous renal replacement therapies. Am J Kidney Dis. 2002;40:275–279. 12148099 10.1053/ajkd.2002.34505

[37] Schiffl H, Fischer R. Five-year outcomes of severe acute kidney injury requiring renal replacement therapy. Nephrol Dial Transplant. 2008;23:2235–2241. 10.1093/ndt/gfn182 18408072

[38] Stads S, Fortrie G, van Bommel J, Zietse R, Betjes MG. Impaired kidney function at hospital discharge and long-term renal and overall survival in patients who received CRRT. Clin J Am Soc Nephrol. 2013;8:1284–1291. 10.2215/CJN.06650712 23599403 PMC3731903

[39] Schiffl H. Renal recovery from acute tubular necrosis requiring renal replacement therapy: a prospective study in critically ill patients. Nephrol Dial Transplant. 2006;21:1248–1252. 16449291 10.1093/ndt/gfk069

[40] Uchino S, Kellum JA, Bellomo R, Doig GS, Morimatsu H, Morgera S, et al Acute renal failure in critically ill patients: a multinational, multicenter study. Jama. 2005;294:813–818. 16106006 10.1001/jama.294.7.813

